# Analysis of Somatic Mutations in the TCGA-LIHC Whole Exome Sequence to Identify the Neoantigen for Immunotherapy in Hepatocellular Carcinoma

**DOI:** 10.3390/cimb46010009

**Published:** 2023-12-22

**Authors:** Swetha Pulakuntla, Khajamohiddin Syed, Vaddi Damodara Reddy

**Affiliations:** 1 School of Applied Sciences, REVA University, Bangalore 560064, Karnataka, India; swethabiochem@gmail.com; 2 Department of Biochemistry and Microbiology, Faculty of Science, Agriculture and Engineering, University of Zululand, KwaDlangezwa 3886, South Africa

**Keywords:** liver hepatocellular carcinoma, somatic mutations, neoantigens, immune checkpoint inhibitors, immunotherapy

## Abstract

There are numerous clinically proven methods for treating cancer worldwide. Immunotherapy has been used to treat cancer with significant success in the current studies. The purpose of this work is to identify somatically altered target gene neoantigens and investigate liver cancer-related immune cell interaction and functional changes for potential immunotherapy in future clinical trials. Clinical patient data from the Cancer Genome Atlas (TCGA) database were used in this investigation. The R maf utility package was used to perform somatic analysis. The 17-mer peptide neoantigens were extracted using an in-house Python software called Peptide.py. Additionally, the epitope analysis was conducted using NetMHCpan4.1 program. Neopeptide immunogenicity was assessed using DeepCNN-Ineo, and tumor immune interaction, association with immune cells, correlation, and survival analysis were assessed using the TIMER web server. Based on somatic mutation analysis, we have identified the top 10 driver genes (TP53, TNN, CTNNB1, MUC16, ALB, PCLO, MUC4, ABCA13, APOB, and RYR2). From the superfamily of 20 HLA (Human leukocyte antigens) allele epitopes, we discovered 5653 neopeptides. Based on T cell receptor face hydrophobic analysis, these neopeptides were subjected to immunogenicity investigation. A mutation linked to tumor growth may have an impact on immune cells. According to this study’s correlation and survival analysis, all driver genes may function as immune targets for liver cancer. These genes are recognized to be immune targets. In the future, immune checkpoint inhibitors may be developed to prolong patient survival times and prevent hepatocellular carcinoma (HCC) through immunotherapy.

## 1. Introduction

Approximately 1.80 billion cases and 830,000 fatalities from liver cancer were predicted for 2020; by 2025, more than 1 million people may be affected [1,2,3]. Liver cancer is currently the third most common cause of cancer-related deaths globally. Out of the total liver malignancies, 85–90% of them are hepatocellular carcinomas (HCC). Chronic hepatitis B and C virus infections include a number of significant risk factors, the most dangerous of which is cirrhosis [4,5]. As the pathogenic co-factors in HCC, alcohol and tobacco are additional related risk factors [6].

Depending on the etiologies and gene alterations, different pathogenic molecular studies have been conducted [7]. Molecular parthenogenesis could be used to identify the disease’s underlying mutations, but the therapeutic use of this newfound understanding is still far in the future [8]. Finding driver genes with oncogenic and suppressive properties in HCC may be improved by the high throughput gene sequence [9]. Telomerase activation mutations, viral insertions, chromosomal changes, and gene duplications are characteristics that set HCC apart [10]. Novel proteins (neoantigens) or tumor-specific proteins that are attached to major histocompatibility complexes on the cell surface and that can be recognized by T cell receptors (TCRs) for additional cell response may be generated by mutations and viral oncogenes [11].

The immune classification of liver cancer has been established by numerous investigations utilizing biological, immunological, genomics, and epigenomics techniques [12,13]. The phrases immune-active, immune-exhausted, and immune-classification were utilized in our study, which was immune-classification oriented. Tumor microenvironment activation of immune-exhausted tumors is a major factor in HCC. It has an increased concentration of helper T(CD4+) cells, and it may cause cytotoxic T (CD8+) cells to react negatively to immune checkpoint inhibitors (ICIs) [14]. The immune system’s current reaction to a tumor attack is known as the ICI, which activates T cells and has demonstrated increased effectiveness in treating a number of solid tumors [12]. For the treatment of liver cancer, the Food and Drug Administration (FDA) has approved ICIs such as ipilimumab, nivolumab, pembrolizumab, and atezolimumab [15,16]. These immune checkpoint inhibitors (ICIs) target T cell immunoglobulin and mucin domain –3 (TIM3), lymphocyte activation gene 3 (LAG-3), programmed cell death protein-1 (PD-1) and ligand (PDL-1), and cytotoxic T-lymphocyte protein-4 (CTLA4). ICIs can shrink tumors and increase survival rates by reactivating repressed T cells that attack cancer [17,18]. Checkpoint blockade therapy is successful, although only a small percentage of patients benefit from it. There are currently no immunological targets that can be used to predict a patient’s response. The identification of unique and uncommon cancer antigens as well as co-inhibitory signaling molecules that coordinate T cell immunotherapy thus constitutes the novelty of this work.

## 2. Materials and Methods

### 2.1. Data Collection

The TCGA database (accessed on 15 July 2022, https://portal.gdc.cancer.gov/) provided the whole exome sequencing (WES) open-source data for LIHC. The mutation annotation format (MAF) file containing all of the patient’s clinical data was obtained. With the Illumina HiSeq 2000 Whole Exome Sequencing Platform, 358 LIHC patients’ sample sequencing was completed. All patients with hepatocellular carcinoma had the liver and intrahepatic bile ducts as their primary sites of cancer. The MAF files were analyzed using the R maftools and TCGA bio links packages.

### 2.2. Identification of Neoantigens

Protein sequences for the top 10 driver genes (P53, TNN, CTNNB1, MUC16, ALB, PCLO, MUC4, ABCA13, APOB, and RYR2) were obtained from the Uniport database (https://www.uniprot.org/, accessed on 15 July 2022). The 17-mer peptide length, where the mutated type (MT) amino acid was in the middle of the other eight amino acids from upstream and downstream, and wildtype (WT) amino acid sequences for the top 10 genes (S2) were extracted using our proprietary Python script (Peptide.py) (Table S1) with pVAC-seq (v.4.08) as the reference [19]. Artificial neural networks are used by NetMHCpan v4.1 software, Lyngby, Denmark to train an epitope analysis algorithm. We selected a superfamily of HLA class-I 20 alleles (HLA-A*01:01, HLA-A*02:01, HLA-A*02:03, HLA-A*02:07, HLA-A*03:01, HLA-A*11:01, HLA-A*24:02, HLA-A*29:02, HLA-A*31:01, HLA-A*32:01, HLA-A*68:02, HLA-A*07:02, HLA-B*15:01, HLA-B*35:01, HLA-B*40:01, HLA-B*44:02, HLA-B* 44:03, HLA-B*51:01, HLA-B*54:01, and HLA-B*57:01) from earlier research [20]. For epitope analysis, the top 10 driver genes were tested against 20 alleles. The key selection and filtering mechanism for all of the peptide binding affinity of MHC (major histocompatibility) molecules is the 9-mer amino acid chain. For this investigation, we selected 9-mer peptides determined by inhibitory concentration (IC50). These IC50 values are assumed to have 500 nM for weak binding and 50 nM for strong binding [21,22,23].

### 2.3. Potential Neoantigen Analysis

The class-I HLA neoantigens’ immunogenicity were predicted by the DeepCNN-Ineo (accessed on 15 July 2022, http://119.3.70.71/dbPepNeo2/deepcnn-ineo.html) based on the score. This application is based on a convolutional neural network-based deep learning model that was generated utilizing curated MHC-I epitope data from the Immune Epitope Database (accessed on 15 July 2022, IEDB, https://www.iedb.org/). The recommended score for high immunogenicity is 0.8, with 0.5–0.8 for low immunogenicity and less than 0.5 for non-immunogenicity.

### 2.4. Immune Profile Studies with Timer Web Server

The tumor immune estimation resource (TIMER) is a web resource for systematic evaluation of the clinical impact of different immune cells in diverse cancer types that (accessed on 15 July 2022, https://cistrome.shinyapps.io/timer) may be used to analyze the relationship and survival analysis between immune gene markers and liver cancer, as well as to determine the infiltrating status for six immune cell types: B cells, CD8+ T cells, CD4+ T cells, macrophages, neutrophils, and dendritic cells [24].

### 2.5. Statistical Analysis

All patient somatic mutation analysis was conducted using statistical significance analysis, and presentations were performed using R v4.0. Fisher’s exact test was used to compare categorical variables. We used Spearman’s correlation and statistical significance to evaluate the correlation of gene expression. The Cox proportional hazards regression model was used to assess the risk factors in the overall survival analysis.

## 3. Results

### 3.1. LIHC Data and Clinical Information Selection

**Figure 1:**
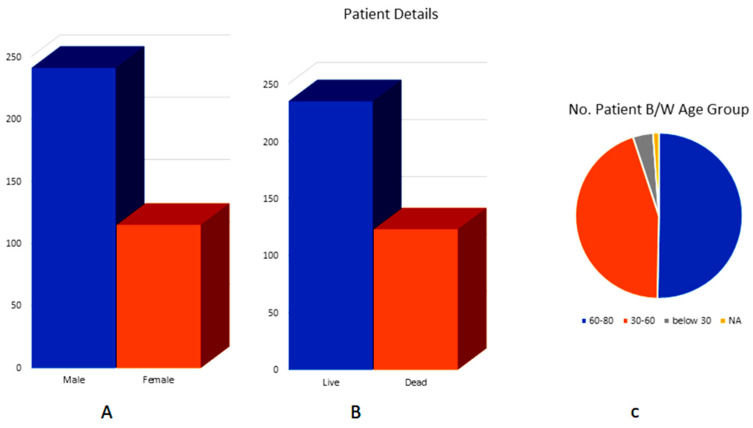
Clinical information on the included LIHC-TCGA samples (**A**) of gender differences between male and female LIHC patients, (**B**) survival status, whether life or death, and (**C**) no. of patients in each age group.

We obtained LIHC data for 358 patient samples including normal and tumor samples from the TCGA using clinical information. Whole exome sequencing (WES) data were used for all of these samples. As seen in Figure 1, this clinical data included the patient ID, gender, age, and survival status. The number of male patients (241) was more than the number of female patients (117) in this instance (Table S2). Using Fisher’s exact test, the death rate in the late stage was considerably high (*p* = 0.0436). The overall survival (OS) of males and females differed slightly.

### 3.2. Analyzing the MAF File for the Somatic Mutation Analysis

**Figure 2:**
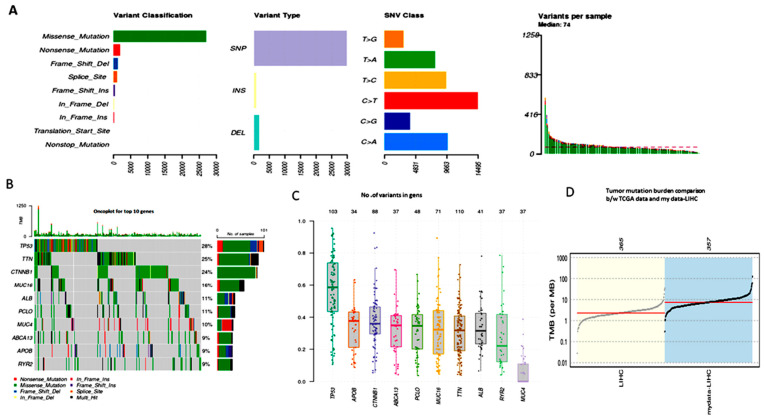
Somatic variant analysis of TCGA-LIHC data. (**A**) Variants per sample in stacked barplot, with variant types as a box plot; (**B**) onco plot for the TOP 10 driver genes; (**C**) driver genes variant allele frequency in the box plot; (**D**) comparison of the mutation load against the TCGA cohorts.

High-level platforms include the full exome sequencing, TCGA-LIHC MAF file data-specific variant calling with MuSE or MuTech. We utilized the R package maftools to statistically summarize and visualize the mutation study. The TCGA sample barcode in the MAF file may be used to identify somatic mutations and determine the frequency of mutations for each patient’s suggested clinical data. Plot maf was utilized to show the variation categorization and kind in a boxplot, with the number of variants in each sample in a stacked barplot. The summary of multiple hits, annotated variants, and mutated genes is shown in Figure 2A. The number of variants in each gene divided by the total number of patients (358) with at least one mutation identified provides the frequency of the genes. Of the 358 total samples in Figure 2B, the top 10 genes altered 268 samples, or 74.86% of the total. The functional plots in boxplot Figure 2C indicate the number of variations in allele mean frequency 50 for each sample, which may help identify the most important driver genes (P53, TNN, CTNNB1, MUC16, ALB, PCLO, MUC4, ABCA13, APOB, and RYR2).

### 3.3. TMB (Tumor Mutational Burden)

The liver cancer mutation landscape has been revealed by NextGen Sequencing (NGS) technology. The TMB measures assess how many non-synonymous somatic mutations there are in each patient’s sample per million base pairs. Previous studies have demonstrated that TMB reacts to solid tumors and may be used to target liver cancer biomarkers with immune treatment. The TMB determines how many mutations there are in each megabase (log10per) of the genomic sequences. It is believed that the TMB is a major factor in the production of immunogenic neopeptides. One sample had no mutations, making the total 358 samples’ mutation burden one. Figure 2D shows a plot of the data, which are the 357 LIHC samples compared with 363 TCGA cohorts.

### 3.4. Mutational Signatures

**Figure 3:**
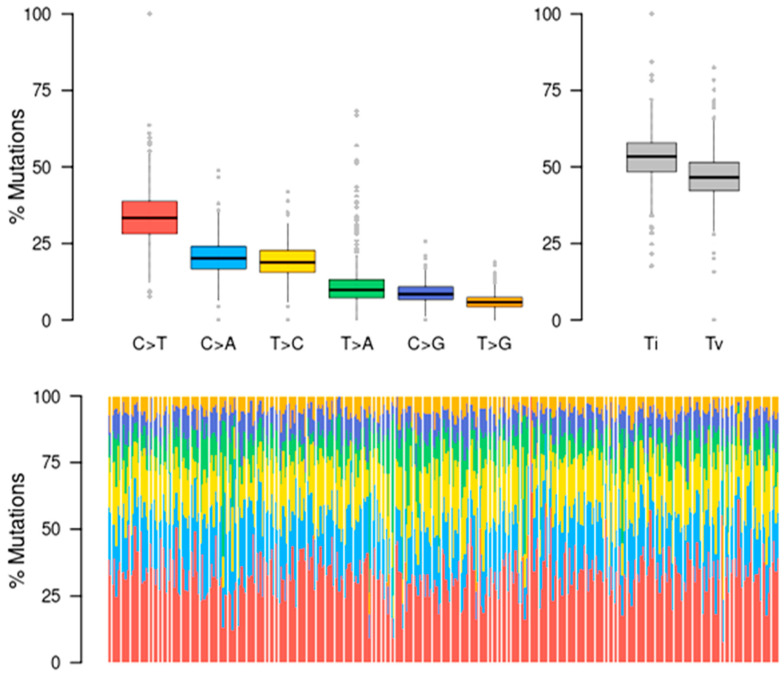
Mutational signatures of liver cancer patients. The functional classification of SNPs is summarized; statistical data were visualized as box plots, with the overall distribution of six different conversions of transitions and transversions.

Most hepatocytes constantly accumulate several DNA mutations and epigenetic modifications along with other risk factors when liver disorders arise. Six DNA substitutions (C > T, C > A, T > C, T > A, C > G, T > G) were found by the somatic mutation analysis of LIHC. Additional classifications of the single nucleotide polymorphisms (SNPs) into transitions (Ti) and transversions (Tv) are displayed in the stacked bar graph in Figure 3. The C > T transition has the highest number of base mutations, but transversions in the C > A and T > C transitions also have the highest number of base mutations. Notably, there are significantly fewer transversions in the base pairs T > A, C > G, and T > G. Transversions, on the other hand, encompass far more than just transitions.

### 3.5. Pathways of Oncogenic Signaling

**Figure 4:**
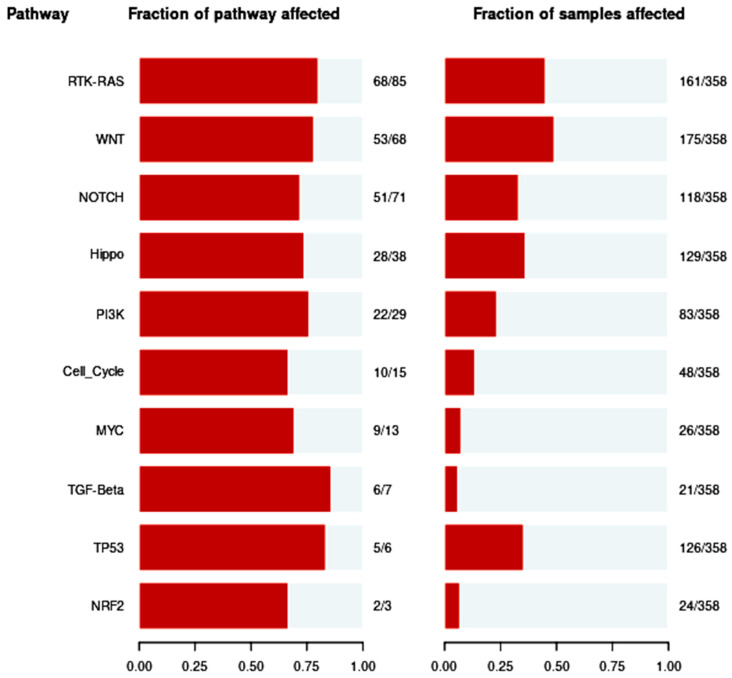
Enrichment of known oncogenic signaling pathways in TCGA-LIHC cohorts.

In the present study, ten well-known signaling pathways such as the RTK-RAS, WNT, NOTCH, Hippo, PIK3, Cell Cycle, MYC, TGF-, TP53, and NRF2 were examined. In addition, we examined the processes behind these somatic alterations. Here, using 358 TCGA-LIHC samples with a fraction of mutations in clusters on the X-axis, as displayed in Figure 4, we worked on a framework to design routes evenly. Numerous studies have shown that TCGA-LIHC commonly alters a wide range of significant pathways. More changed pathways, such as 80% RTK-RAS or cell-cycle pathways in numerous tumor types, are also present in tumors with the highest tumor mutation burden. In total, 30% to 50% of the Wnt signaling pathways are caused by the CTNNB1 gene mutation. In 70% of other driver genes, P53 genomic alterations changed gene-centricity as well as intra- and inter-pathway interactions. This pathway particularly responds to immune checkpoint inhibitors and may be enhanced by CD4+ and CD8+ cell infiltrations and immunological categorization of HCC tumors. It might help in the treatment of cancer.

### 3.6. Identification of Neoantigens (Peptide Selection and Epitope Analysis)

To choose the 17-mer peptide length MT and WT types in FASTA format, we created a bespoke Python tool (Table S3). For the epitope analysis, we selected the top 10 somatic mutation driver genes from the TCGA-LIHC. The Uniport database was the source of the driver gene protein sequence. We chose super family HLA-Class-I (20) alleles, enabling us to provide results that may account for 95% of the world’s human population. Additionally, using the elution ligand method (EL) and the academically licensed NetMHCpan-4.1 software, epitope analysis was carried out using the HLA alleles versus peptides as inputs. A large amount of allele training data was used to accurately quantify the peptide’s prediction of the HLA binding affinity. On the basis of the IC50 threshold (500 nM) values, we were able to forecast all potential mutant epitopes by strong and weak binding affinity. Since 9-mer peptides may comprise 90% of neoantigens, we exclusively took them into consideration. Human T cell responses may demonstrate this.

**Figure 5:**
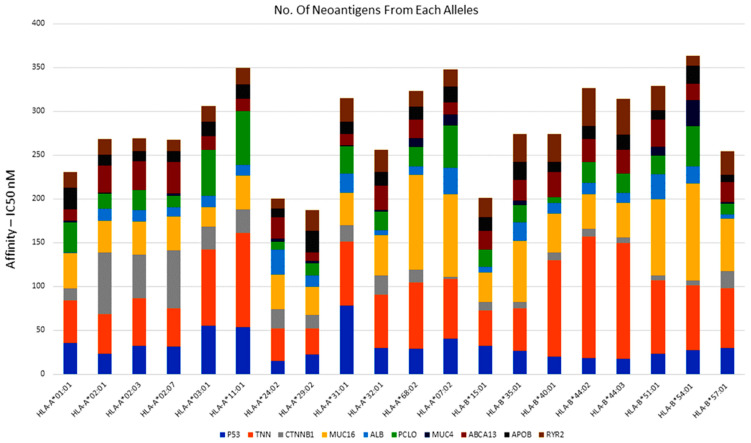
High throughput prediction of MHC class-I alleles: the neoantigen-specific prediction based on IC50 binding affinity (IC50 < 500 nM) and the top mutated genes are indicated with different color bar graphs.

Using the default NetMhcpan4.1 IC50 values, we filtered 5653 neopeptides of driver gene missense mutations (Table S4). The altered peptides are recognized by cytotoxic CD8+ T cells when they bind to class-I MHC molecules and nucleotides. Following the expected extension and characterization of the investigation, there appeared to be a correlation between the observed number of neopeptides and the mutation burden. Figure 5 shows the findings of the largest amount of neopeptides’ predicted frequency driving genes.

### 3.7. Identification of Potential Neoantigens

**Figure 6:**
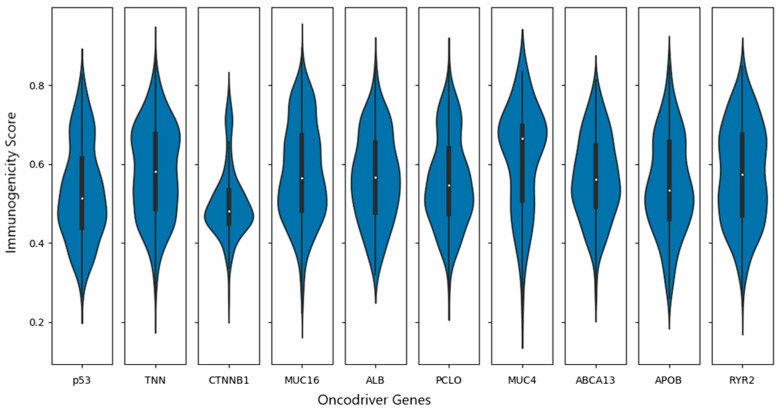
Immunogenicity of recurrent oncogenic genes non-synonymous mutations’ distribution shown in Violin plots. The points show the median values, where 0.8 indicates high immunogenicity, 0.5–0.8 means low immunogenicity, and less than 0.5 is non-immunogenic.

Ultimately, neoantigens and epitopes from the NetMHCpan study were confirmed by HLA-class-I 8–9 amino acid length. Because they are more sensitive, a few amino acids choose to add N- and C-terminal anchor sites. TCR binds to hydrophobic amino acids most of the time. Epitopes with this frequency are very immunogenic. The frequency of the neopeptide immunogenicity features was validated in the IEDB based on the charge. To predict the TCR epitopes for both immunogenic and non-immunogenic neopeptides, we employed the website DeepCNN-Ineo program. To identify neoantigens using the top 10 driver genes, we employed nine superfamilies (20) of HLA class-I alleles (Table S5). The suggested peptide length is eight or twelve amino acids, with position two at the N-terminus and position C-terminal being used as the terminal anchor sites by default. The prediction scores were less than 0.5 when contemplating non-immunogenicity (negative high), higher than 0.5 when considering high positive immunogenicity, and between 0.5 and 0.8 when indicating low positive immunogenicity. These values show that the immunogenicity score is indicated on the Y-axis of all neoantigen immunogenicity values that were plotted using the Python script results. Every driver gene with 20 HLA alleles on the X-axis is a neoantigen (Figure 6).

### 3.8. Immune Profile Data Analysis

**Figure 7:**
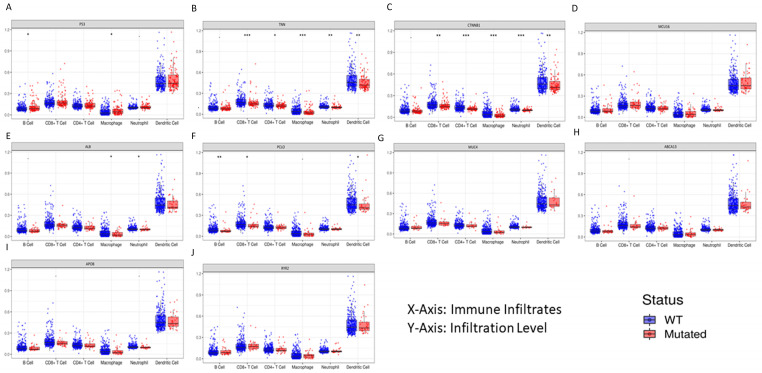
The box plots from the mutation module display the difference in the tumor immune estimation resource (TIMER)-estimated in six immune cell (B Cell, CD8+T Cell, CD4+ T Cell, macrophage, neutrophil, and dendritic cell) infiltrates level estimated between mutant and wildtype driver genes (**A**). TP53, (**B**). TNN, (**C**). CTNNB1, (**D**). MUC16, (**E**). ALB, (**F**). PCLO, (**G**). MUC4, (**H**). ABCA13, (**I**). APOB, and (**J**). RYR2, respectively, in liver cancer.

Genetic changes are linked to the growth and spread of tumors, and these changes may have an impact on the immune cells that infiltrate tumors (TIIC). All six immune cell types—B cells, CD8+ T cells, CD4+ T cells, macrophages, neutrophils, and dendritic cells—in their WT and mutant states could be compared with the driver gene somatic mutation. P53 (28%), TNN (25%), CTNNB1 (24%), MUC16 (16%), PCLO (11%), ALB (11%), MUC4 (10), ABCA13 (9%), APOB (9%), and RYR2 (9%) are the 10 most frequently mutated genes. With the use of the dynamic web interface tool TIMER, the mutation module was used to examine the immunological infiltration. The box plots produced for each immune group are shown in Figure 7, where the immune infiltration distribution level of each gene mutation is compared using statistically significant values and a 95% confidence interval, determined using the two-sided Wilcoxon rank sum test.

### 3.9. Investigate Immune Checkpoint Inhibitors

**Figure 8:**
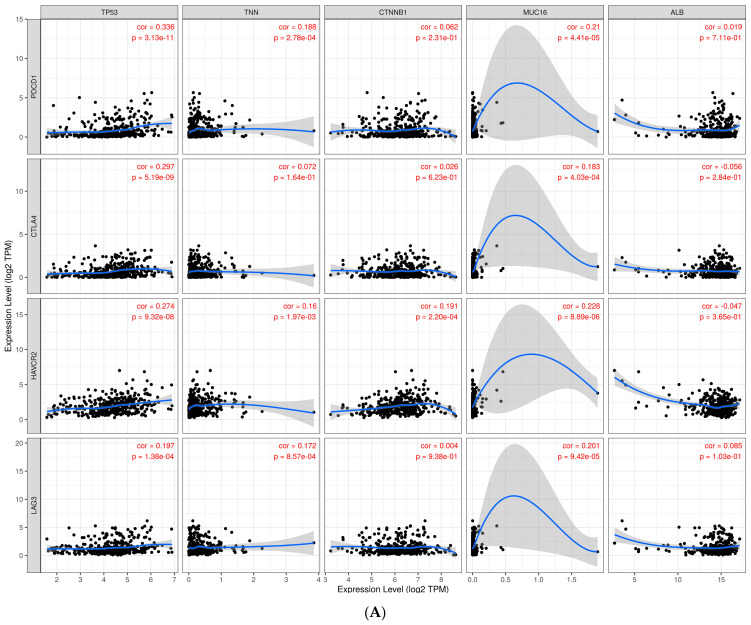

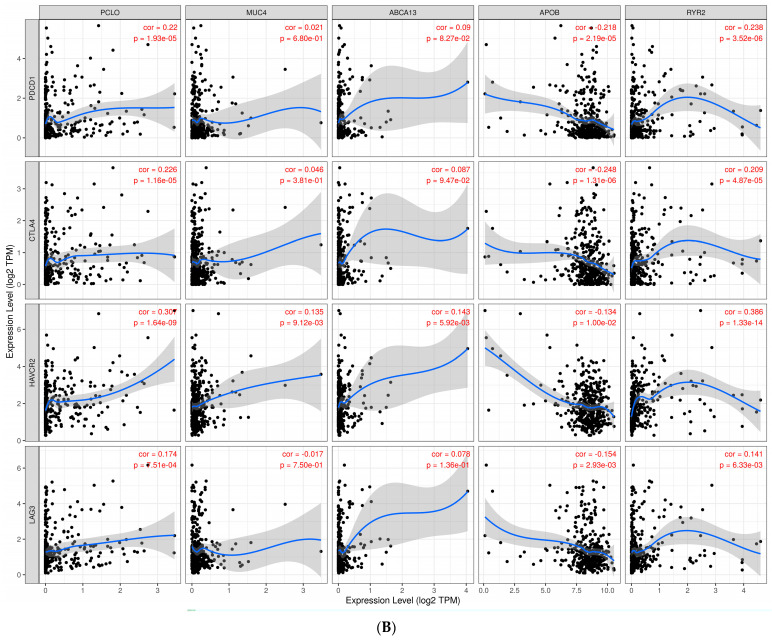
(**A**). Scatter plots derived from the relationship between the expression of driver genes (TP53, TNN, CTNNB1, MUC16, and ALB) on the X-axis and known immune targets (PDCD1, CTLA4, HAVCR2, and LAG3) on the Y-axis. (**B**). Scatter plots derived from the association between driver genes (PCLO, MUC4, ABCA13, APOB, and RYR2) and known immunological targets (PDCD1, CTLA4, HAVCR2, and LAG3) on the X- and Y-axes.

Immunotherapy using immune checkpoint inhibitors is currently the most successful therapeutic treatment for metastatic liver cancer. This is an important development in cancer biomarker prediction. In this work, we examined the relationship between immune cells and tumor immune infiltration, and we found that immune inhibitory receptors such as PDCD1, CTLA4, TIM3, and LAG3 may be important for T cell activation in tumor cells. Dostarlimab (TSR-042), an antibody that blocks the PDCD1 receptor, was recently approved by the FDA to treat endometrial cancer. Advanced solid tumors have been used in this clinical trial (NCT02715284) to test the antibody mismatch repair and DNA repair functions [25]. There are ongoing phase-II clinical trials (NCT03680508) using TSR-042 PDCD1 (PD-1) and TSR-022 anti-HAVCR2 (TIM3) antibodies for primary liver cancer. We examined the computational relationship between the widespread ICB receptors PDCD1/HAVCR2 and LAG3 and the TCGA-LIHC driver gene. According to earlier research, CD8+ T cells are associated with inhibitor receptors, which trigger T cell activation for therapeutic liver cancer treatment. Not on a normal liver tissue sample but on CD8+ T cell malignancies, elevated expression of TIM-3 and LAG-3 may facilitate immune evasion and poor prognosis. The TIMER analysis revealed that PDCD1, CTLA4, HAVCR2, and LAG-3 correlated with all ten of the TCGA-LIHC’s top genes, with correlation values ranging from 0.19 to 1. The significant *p* values for this statistical connection, which were determined using Spearman’s rho value, are displayed in Figure 8A,B.

**Figure 9:**
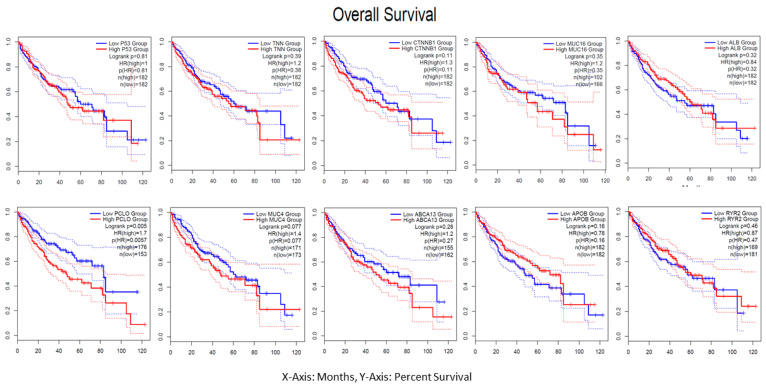
The overall survival analysis plot shows the median values on the Y-axis and months on the X-axis, indicating all driver genes TP53, TNN, CTNNB1, MUC16, ALB, PCLO, MUC4, ABCA13, APOB, and RYR2, respectively.

The median group computed the hazard ratio (HR) on the Cox PH model with a 95% confidence interval (CI) by analyzing the TCGA-LIHC of each driver gene’s overall survival. Red denotes a high group risk, and blue denotes a low group risk in the group survival plot displayed in Figure 9. An examination of the tumor mutation burden survival shows a correlation between these driver genes. Future immunotherapy research, including ICI clinical studies, will be necessary.

## 4. Discussion

The study group is gradually developing next-generation sequencing technology to combine massive amounts of data for immune therapy-related mutation studies in various malignancies. The entire exome sequences of 358 patients’ sample maf files, which were obtained from the TCGA database, were used in the current investigation. Using R maf tools, we conducted a mutation study and discovered a large number of mutant genes. The number of neoantigens with somatic mutations per megabase in cancer correlates with the tumor mutation burden [26]. Using the netMHCpan4.1 program, an epitope analysis was conducted on a modified peptide sequence against 20 superfamily HLA-I alleles. Based on the IC50 values of 500 nM strong interaction with T cells, MHC molecules were predicted for the 8–9 mer epitopes/neoantigens. We identified possible neoantigens based on the immunogenicity score of the neopeptides using the DeepCNN-Ineo website. Data from the IEDB repository were used to train this program. The CD8+ T cells were classified as immunogenic or non-immunogenic based on the relative hydrophobicity of amino acids in this study. The T cell receptors could identify the antigenic character of the epitopes. With low G+C genomic codons potentially having an impact on the amino acid use case, the majority of the data included in this study came from intracellular diseases such as viruses [27]. These more hydrophobic potential pathogens may be employed to identify T cell receptors. Based on their close proximity to antigens, hydrophobic areas have been found to greatly boost the rate of proteasomal breakdown and the immunogenic T cell epitopes [28].

Current research on immunotherapy is leading to a greater understanding of how the immune system infiltrates cancer. It is difficult to analyze and visualize the vast amounts of clinical and genetic data. Developing a unique computational technique to deconvolve complex data is essential to investigate tumor–immune interactions [29]. Using six immune cells—B cells, CD8+ T cells, CD4+ T cells, macrophages, neutrophils, and dendritic cells—we comprehensively analyzed the immunological and genomic characteristics of tumors in our study using the TIMER online server-based technology [30]. Mutations in the top driver genes identified by TCGA-LIHC (TP53, TNN, CTNNB1, MUC16, ALB, PCLO, MUC4, ABCA13, APOB, and RYR2) were examined. Box plots were constructed for each immune cell subgroup, and the expression displayed the statistically significant values that were assessed using the two-sided Wilcoxon rank sum test (Figure 7). We found that CD8+ immune infiltration enhanced the tumors with high mutant gene counts in all TCGA-LIHC patients. We postulated that the immunogenic mutations in these predictions may reflect the expression of CD8+ [31].

The relationship between liver cancer driver genes and immune cell receptors was analyzed using TIMER in another module, and the results indicated a modest correlation with Spearman’s statistical significance (0.9–1). The most significant immune biomarkers for hepatocellular carcinoma were employed in immunotherapy, according to earlier research: PDCD1, CTLA4, HAVCR2/TIM3, and LAG3 [32]. Liver cancer patients now have access to immune checkpoint inhibitor therapy. Both the tumor cells and the surrounding environment should be affected by the immunotherapy [33]. The immune response of CD8+ immune cells and the PDCD1 tumor association may be the targets of ICI molecules, which can also indicate the prognosis of cancer. TIM3 and LAG3 receptors are present on CD4+ and CD8+ immune cells [34]. Hepatocytes manufacture a unique functional fibrinogen-like protein-1 ligand that is exclusive to LAG3. These research preclinical findings back up the exploration of TIM3, LAG-3, and PDCD1 inhibition in liver cancer cases [35].

The best mean survival was 22.8 months based on the initial study of immunological biomarkers (CTLA4, PDCD1) and ICI combinations of ipilimumab and nivolumab [36]. The FDA approved the combinational ICI and additional combinations of durvalumab and tremelimumab for the PDCD1 in light of these positive results [37]. Atemalizumab and bevacizumab were licensed by the FDA for use in immunotherapy for the first HCC combination on a worldwide scale [38]. These findings have led to the present clinical trials of a number of ICI combinations on patients receiving systemic immune treatment. Adoptive cell therapy for liver cancer also involves lymphocyte sensitization therapy, which is carried out in vivo prior to being reinfused into the patient [39]. The cytokine killer cells are triggered by the lymphokine. Another extremely effective and promising treatment for hematological malignancies with solid tumors that is still in the research phase is the CAR T cell [40]. The antigen domain of this treatment is made up of a monoclonal antibody signal fragment that is tailored to a particular tumor cell target. The target cell’s toxicity is one drawback of this CAR T cell treatment. The target cells express themselves in normal cells as a result. GPC3 CAR T cell intravenous injunction is undergoing safe and effective clinical trials (NCT04121273) [41].

The use of vaccines to prevent cancer is another important factor. The potency may rise due to the tumor-specific response. This demonstrates how well T lymphocytes are prepared to combat antigens produced by cancer cells [42]. Historically, vaccinations have been administered as a standalone treatment; however, it is now known that immunosuppression in the tumor microenvironment increases T cell activity [43]. Additional immunological vaccinations may detect the antigen specific to a tumor. HLA typing is the best method to use for immune-related peptide identification in this case. Furthermore, specific immunological signatures associated with cytotoxic T-lymphocytes (CTLs) are expressed along with this HLA peptidome. Based on these immunological signature results on board, these vaccination clinical studies for liver cancer are still ongoing [44].

## 5. Conclusions

It is necessary to expand the gene mutation analysis from the sequence source in this investigation of immunotherapy for liver cancer. Novel neoantigens unique to tumor antigens recognized by T lymphocytes may be found as a result of somatic mutations. Further research on immune cell profiles and immune infiltration expression CD8+ predictions are indicated by the altered genes. In order to identify novel immune biomarkers from the TCGA-LIHC and examine associated expression with established immune biomarkers for T cell activation with an immune checkpoint inhibitor in liver cancer, this study examined PDCD1, CTLA4, HAVCR2, and LAG3. These investigations on immune receptors and altered genes may prove useful in the future for developing vaccines and ICIs for the immunotherapy of liver cancer.

## Data Availability

All data are available in the main manuscript and supplementary information.

## Supplementary Materials

The following supporting information can be downloaded at: https://www.mdpi.com/article/10.3390/cimb46010009/s1, Table S1: In-house c program for the 18-mer neoantigen peptide filter from the wildtype and normal peptide sequences; Table S2: XLS file of 358 patients’ clinical information; Table S3: XLS file of the top 10 genes’ 18-mer peptide sequence (wildtype and mutant); Table S4: XLS file of the 18-mer protein sequence with superfamily HLA class-1 alleles using NetMhcpan software extensive data; Table S5: XLS file for the potential neoantigen analysis data.
