# Supplementary material for: Analysis of Somatic Mutations in the TCGA-LIHC Whole Exome Sequence to Identify the Neoantigen for Immunotherapy in Hepatocellular Carcinoma
Source: Curr Issues Mol Biol. 2023 Dec 22;46(1):106–20. doi: 10.3390/cimb46010009 (PMC10813969; doi:10.3390/cimb46010009)
Supplement: Supplementary file 1 [file cimb-46-00009-s001.zip › S1_python script.pdf]

```
# input and out file read and write

fd1 = open("proteinSeq", "r")

fd2 = open("mutationAAC", "r")

fd3 = open("mutateseq.fsa", "w+")

#line defining and counting from the inputfile fd2

Lines = fd2.readlines()

# read mutation one by one

for line in Lines:

    #print ("Lines{}:{}".format (count, line.strip()))

    #print (line.strip())

    mut = line.strip()

    pos = ""

    for i in mut:

        if i.isdigit():

      pos = pos + i

    exist_aa = mut[0]

    mutate_aa = mut[len(mut)-1]

    #print (exist_aa, mutate_aa)

    pos = int(pos)

    pos = pos-1

    #print (pos)

#finding seq position from the fd1 and definding from the mutated position required numbe writting the 17 mer fasta file as wt and mt to mutateseq.fsa

    fd1.seek(pos, 0)

    if (fd1.read(1) == exist_aa):

        fd1.seek(pos-8, 0)

        str = fd1.read(17)

        fd3.write(">WT")

        fd3.write(mut)
```

```
fd3.write("\n")
fd3.write(str)
fd3.write("\n")
str = list(str)
str[9] = mutate_aa
str = ''.join(str)
fd3.write(">MT")
fd3.write(mut)
fd3.write("\n")
fd3.write(str)
fd3.write("\n")
```
